# On thermodynamic inconsistencies in several photosynthetic and solar cell models and how to fix them†
†Electronic supplementary information (ESI) available: (A) Dynamic equations for standar donor–acceptor models; (B) standard FMO models; (C) detailed derivation of dynamic equations for simple models for the RC/circuit. See DOI: 10.1039/c6sc04350j



**DOI:** 10.1039/c6sc04350j

**Published:** 2016-10-26

**Authors:** David Gelbwaser-Klimovsky, Alán Aspuru-Guzik

**Affiliations:** a Department of Chemistry and Chemical Biology , Cambridge , MA 02138 , USA . Email: dgelbwaser@fas.harvard.edu

## Abstract

We analyze standard theoretical models of solar energy conversion developed to study solar cells and photosynthetic systems. We show that assuming the energy transfer to the reaction center/electric circuit is through a decay rate or “sink”, contradicts the second law of thermodynamics. We put forward a thermodynamically consistent alternative by explicitly considering parts of the reaction center/electric circuit and by employing a Hamiltonian transfer. The predicted energy transfer by the new scheme differs from the one found using a decay rate, casting doubts on the validity of the conclusions obtained by models which include the latter.

## Abstract 2

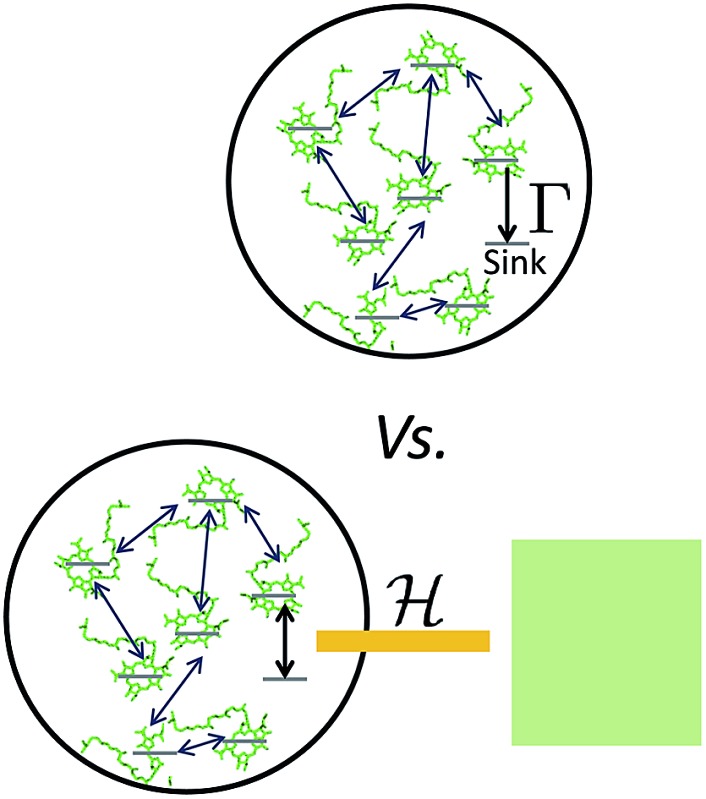
We analyze standard theoretical models of solar energy conversion developed to study solar cells and photosynthetic systems.

Light-harvesting organisms and solar cells convert thermal photons from the Sun, into useful energy such as ATP or electric power.^1–3^ Understanding and improving these processes may lead to more efficient ways to produce clean energy.^4^ These systems are effectively heat engines^5–9^ because they transform a heat flow into power (useful energy). Therefore, they are constrained by the laws of thermodynamics^4,10–16^ which set a fundamental efficiency bound based on the distinction between the two forms of energy exchange: heat flow and power. These two are not interchangeable: in a cyclic process, power may be totally converted into heat flow, but the opposite is forbidden by the second law of thermodynamics.^7,17,18^

A key for understanding the efficiency and the power produced by solar cells and plants, is the development of microscopical models of energy absorption, transmission and storage. Recent researches have shown that effects such as environment assisted quantum transport,^19–22^ coherent nuclear motion,^23,24^ and quantum coherences,^25–28^ play an important role in the enhancement of energy conversion. The importance of some of these effects for energy transport have been experimentally proven in specific realizations such as genetically engineered excitonic networks,^29^ waveguides networks,^30^ Rydberg aggregates^31^ and electrical oscillators.^32^

For practical computational and theoretical reasons, models have been restricted to the study of specific subsystems. It is customary to study photosynthetic complexes coupled to “traps” or “sinks” that represent the reaction center (RC) where exciton dissociation occurs.^19–22,32^ Similar models have been employed for the study of solar cells and exciton absorption and transport.^24–27,33,34^

Here we show that if not careful, the introduction of sinks and traps leads to violations of the second law of thermodynamics. These violations are a reason of concern for the validity of the models that have been employed to date. To shed light on the issue and to provide a simple to understand situation, we introduce a toy model to study this approximation and put forward a thermodynamically consistent version of it. This model could be used as the basis for more elaborate solar cell and plant microscopic models. Finally, we show that the output power of the thermodynamically-consistent version of the model can differ substantially from the simple trap or sink models.

## Second law of thermodynamics and solar energy conversion

**Fig. 1:**
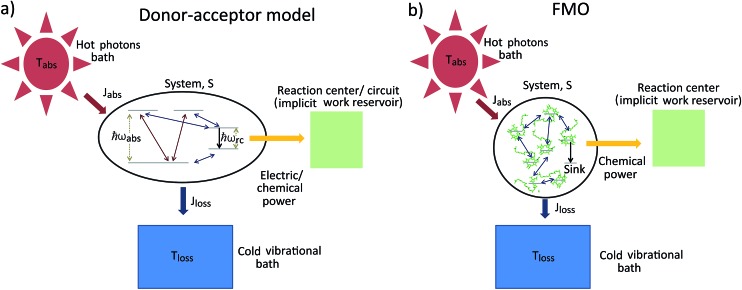
Solar energy conversion models: (a) donor–acceptor model; (b) FMO model. In both cases the allowed transitions are shown only for illustration purposes and may vary between different models.

The standard thermodynamic models for solar energy conversion are comprised by a system, S, that interacts with different thermal baths and transforms the solar energy into chemical energy or electric current. Here we analyze two types of models: donor–acceptor models, where S is composed of four to five levels. These models have been applied for studying solar cells^26,27^ as well as photosynthetic systems,^25^ (see Fig. 1a); a second type of models describe the celebrated Fenna–Matthews–Olson (FMO) complex models, where S includes seven bacteriochlorophyll, each of them described by a single energy state^19–22,24,32–34^ (see Fig. 1b). In both cases, the energy conversion process is composed of the following explicit or implicit steps: (i) *Light absorption*. The system, S, absorbs hot photons coming from the Sun. The temperature of the photon is *T*_abs_ and *J*_abs_ is the heat flow between the hot photons bath and S; (ii) *Energy transfer*. The absorbed energy is transmitted between different states of S. During this stage, some energy is lost through a heat current, *J*_loss_, to a vibrational bath at room temperature *T*_loss_ (material photons for solar cells^2,3^ or protein modes for photosynthetic systems^1^); (iii) *Power extraction*. A decay rate that represents an irreversible energy flow to an external system, work reservoir. The latter is generally not explicitly considered. For photosynthetic models, this last stage involves the decay to a sink or trap, together with an energy transfer to the RC and its subsequent transformation into chemical energy. In the case of solar cells, the energy flow produced by the decay rate is the electric power that runs through the circuit. These models are fundamentally different from the ones used on ref. 29–32, where the sink or trap is explicitly described by a Hamiltonian as we do below.

The dynamics of these systems is constrained by the second law of thermodynamics, through the entropy production inequality,^8,35^
1
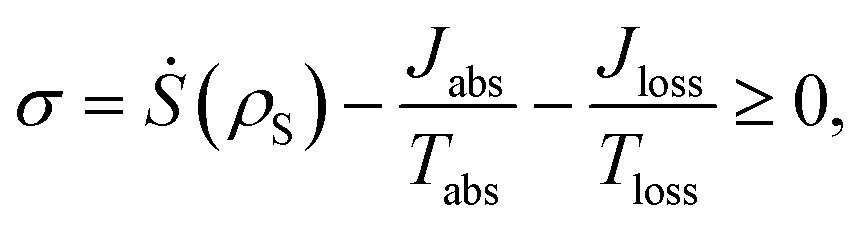
where *σ* is the entropy production, *ρ*_S_ is S density matrix and ** is the derivative over time of the Von-Neumann entropy.^36^ The heat current to the i-bath is the energy flow between S and the bath,^8^

2 *J*_i_ = *Tr*[ℒ_i_(*ρ*_S_)*H*],

where ℒ_i_(*ρ*_S_) is the evolution induced by the i-bath on S or excitation rate, and *H* is the system Hamiltonian which is equivalent to the excitation energy. For the heat currents, as wells as for the power, we use the sign convention that energy flowing to (from) S is positive (negative). Models with artificial sinks could be envisioned as systems that transfer energy to a zero-temperature bath. This could justify the addition of an extra term on the r.h.s of eqn (1) that would allow a 100% energy conversion efficiency. Nevertheless, solar cells and plants must obey the same thermodynamic bound as a heat engine operating between thermal baths at the temperatures of the Sun and the vibrational bath, which are 6000 K and 300 K, respectively, and therefore bounded to 95%. More elaborate models predict an even lower bound.^4,10–16^

In the case of a steady state flux of solar energy into S, the state of S on average does not change, and the second law, eqn (1), simplifies to3
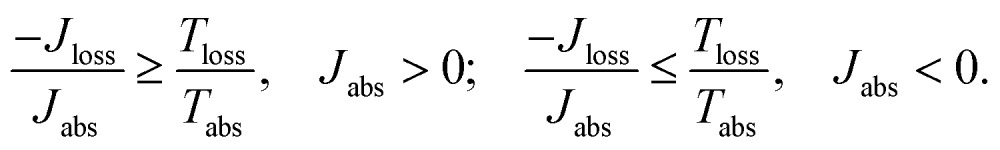



The donor/acceptor models studied in ref. 25–28, analyze the solar energy conversion at steady state. Their heat currents ratio has the form (see ESI-IA†):4
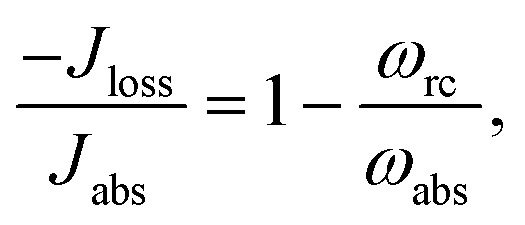
where *ω*_abs_ is the energy of the absorbed photons and *ω*_rc_ is the energy of the excitation transferred to the RC/circuit (work reservoir) (see Fig. 1a). In all these models, the signs of the currents are independent of the parameters, *J*_loss_ < 0 and *J*_abs_ > 0 (see ESI-IA†).

**Fig. 2:**
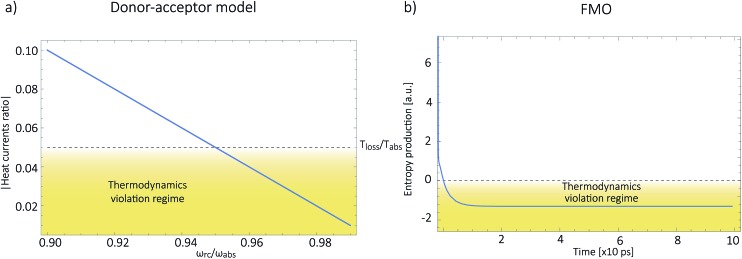
(a) Absolute value of the heat currents ratio as function of frequency ratio for the steady state models in ref. 25–28; (b) entropy production as a function of time for FMO models.^19–22,24,32–34,37^ In both graphs the shaded area represents a regime forbidden by thermodynamics laws.

As shown in Fig. 2a, for 
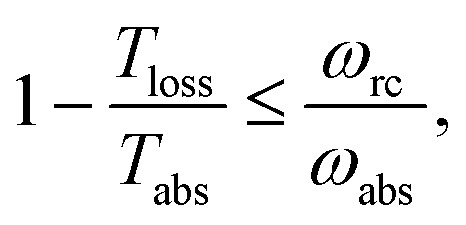
 these models violate the second law of thermodynamics. Realistic model parameters may well fall outside of this range. This does not exclude the fact that the model is both inconsistent and potentially leading to artificial results. As we show below, the power predicted by a thermodynamically consistent model differs from the simple sink or trap models.

Next, we consider the standard sink or trap models of the FMO complex,^19–22,24,32–34,37^ governed by *H*_FMO_ which includes the FMO sites, the vibrational bath and their interaction, as well as the transfer to the RC (see ESI-IB†). The later is a decay term (see Fig. 1b). In order to calculate the entropy production, eqn (1), we include the antenna and the solar thermal radiation.

The antenna is composed of around *N* = 10 000 absorbing pigments,^38^ and we model their collective effect as an effective monochromatic antenna of frequency *ω*_ant_ = 13 333 cm^–1^ or two level system (TLS), with a transition dipole moment 
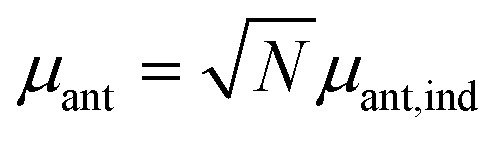
, where *μ*_ant,ind_ ∼ 5 Debye, a typical value for a molecular transition dipole moment. Light absorption is governed by the antenna–radiation coupling, *H*_ant–rad_ = *μ*_ant_|ant0| ⊗ *B*_abs_ + h.c., where *B*_abs_ is an operator on the thermal radiation bath, |ant is the antenna excited state and |0 is the ground state. h.c. stands for the Hermitian conjugate. The FMO sites also interacts with the thermal radiation through the Hamiltonian, 
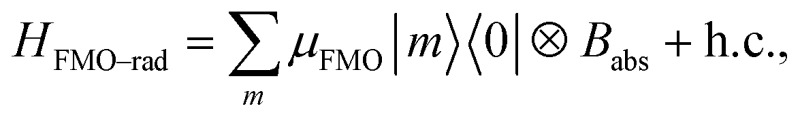
 where |*m* is the excited state of the FMO *m* site and *μ*_FMO_ = 5.44 Debye.^39^

The transmission of the excitation from the antenna to the FMO is assisted by the vibrational degrees of freedom and described by 5
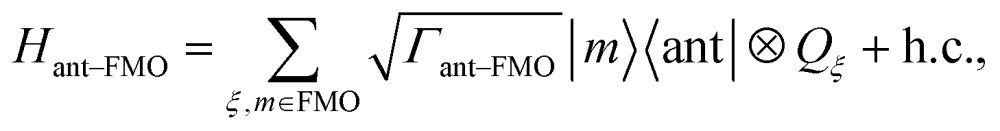
 where *Q*_*ξ*_ operates on the vibrational degrees of freedom. We assume that *Γ*_ant–FMO_ = *Γ*_3,8_/10.

Collecting everything together, the total Hamiltonian is

6 *H*_FMO_ + *H*_ant_ + *H*_rad_ + *H*_ant–FMO_ + *H*_ant–rad_ + *H*_FMO–rad_,

where *H*_ant(rad)_ is the antenna (radiation) free Hamiltonian.

In this scenario, the dynamics outside the steady state is considered. For these models, there is not a simple analytical expression such as eqn (4), therefore we use a standard numeric simulation based on a Lindblad equation^40–42^ to calculate the dynamics governed by eqn (6) and together with eqn (2) we obtain S evolution and the heat currents *J*_abs_(*J*_loss_) between the FMO and the radiation (vibrational) baths. In Fig. 2b the entropy production, eqn (1), as function of time is presented, showing a violation of the second law of thermodynamics.

## Thermodynamically-consistent model

The assumption in the trap or sink models that the energy transfer to the RC/circuit is based solely on a relaxation process, introduces an inconsistency with thermodynamics, which is independent of the trap temperature, chemical potential or if it is modeled as an absorbing boundary by a non-Hermitian Hamiltonian. As it was shown in ref. 43, the later can be reformulated as an open quantum system. Even though physically the energy flow to the RC/circuit is power, a decay rate, or an absorbing boundary, effectively represents a heat flow. This is the root of the inconsistency. Here we use a toy model to clarify this point and put forward an alternative that could serve as basis to correctly model these systems. We compare between two possible energy transfers schemes to the RC/circuit: (i) standard decay; (ii) Hamiltonian transfer.

**Fig. 3:**
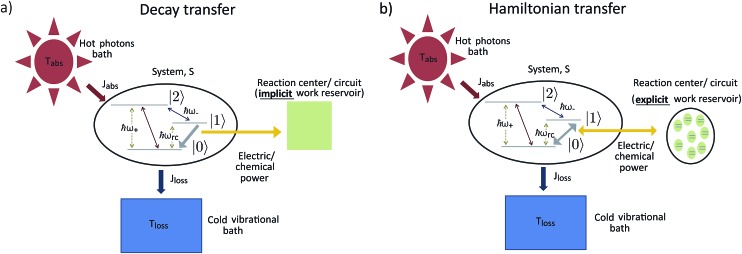
A toy model used to study different energy transfer schemes: decay rate (left); Hamiltonian transfer (right).

For both schemes, S is a three level system as shown in Fig. 3. The absorption of a photon causes an excitation transfer between |0 and |2, whereas phonons are emitted by transitions from |2 to |1 to the vibrational bath. Finally, the cycle is closed by a transition between |1 and |0, and the energy difference is transferred to the RC/circuit. The S-bath Hamiltonian is *H*_S_ + *H*_B_ + *H*_SB_, where *H*_B_ includes the photon and vibrational bath free Hamiltonian. Both baths are in thermal equilibrium at temperatures *T*_abs_ and *T*_loss_, respectively. The S Hamiltonian is7
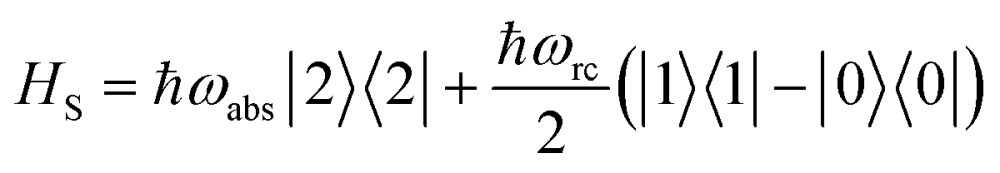
and the S-bath interaction is8
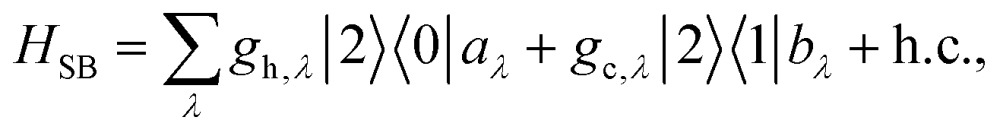
 where *a*_*λ*_,*a*†*λ* (*b*_*λ*_,*b*†*λ*) are the annihilation and creation operator of photons (phonons) modes.

(i) *Decay transfer*. The standard relaxation scheme is a decay rate between |1 and |0,9
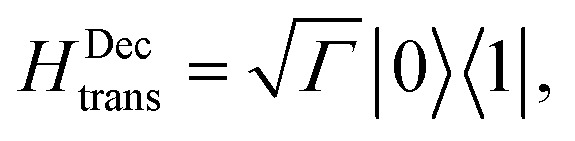
where the RC/circuit is not explicitly included;

(ii) *Hamiltonian transfer*. An alternative to the model above is to explicitly include at least part of the RC/circuit, which plays the role of the work reservoir. In photosynthetic systems, the last stage on the reaction center is the transfer of electrons to the *Q*_B_ quinone, that once full, migrates to drive the production of ATP.^44^ This quinone is replaced by an empty one from a quinone pool. Inspired by this process, we construct a toy model of the work reservoir that could be a guideline for more complicated photosynthetic or solar cells models. It consists of a collection of independent and identical TLSs. Each of them represents a quinone in a photosynthetic system or an electrode site in a solar cell. The ground state corresponds to an empty quinone/site, and the excited state to a “full” quinone/site. Furthermore, we assume that there are always empty quinones/sites available to accept an electron. Thus, the number of quinones/sites, *j*, is always much larger than the number of electrons *c* ^†^ *c*, *J* ≫ *c* ^†^ *c*. This assumption is equivalent to the thermodynamic limit taken in the Holstein–Primakoff procedure,^45,46^ which allows to describe the collection of quinones/sites as a single harmonic oscillator (HO). Therefore, we can write the work reservoir and transfer Hamiltonian as (see ESI-IIB†)10
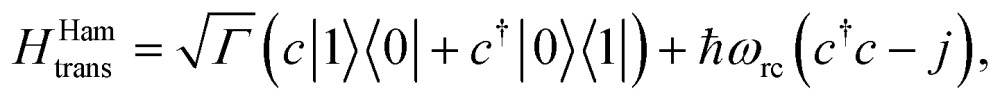
where *c*, *c* ^†^ are the annihilation and creation operators of the HO, respectively. The use of a HO as work reservoir is a common feature of self contained thermal machines and has been broadly studied in the context of quantum thermodynamics and optomechanical devices.^17,18,47–49^ The S + HO Hamiltonian, eqn (7) and (10), is diagonal in a dressed state basis and takes the following form11
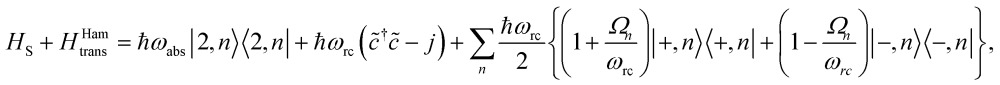
where *c* ^†^(*c*) is the creation (annihilation) operator in the new basis, 
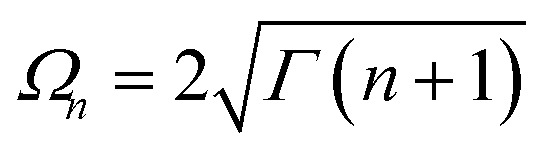
 and12
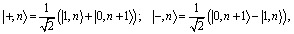
where the first index in the bras and kets refers to the three level system and the second to the HO.

In both schemes we use the standard Born–Markov approximation^36^ and write the Lindblad equations for (see ESI-II†): (i) *Decay transfer scheme*. The three level system, whose populations are given by *ρ*_*i*_ where *i* ∈ {0, 1, 2}; (ii) *Hamiltonian transfer scheme*. The three level system and the HO, whose populations are given by *ρ*_*i*,*n*_ where *i* ∈ {–, +, 2}. For both schemes, we analyze the energy transfer at the three level system steady state and assume that the zero temperature decay rates^50^ of the baths are equal to the transfer rate to the RC/circuit, *Γ*_h_ = *Γ*_c_ = *Γ* (see ESI-II†).

(i) *Decay transfer*. For this case the evolution equations in the interaction picture are (see ESI-IIA†)13
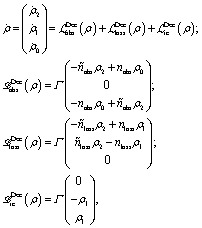
where *n*_abs(loss)_ is the photon (vibrational) bath population of mode 
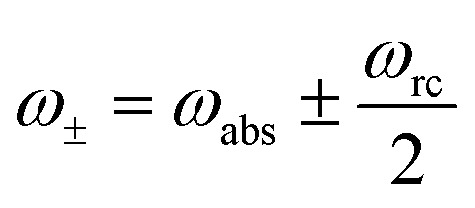
 and *ñ*_abs(loss)_ = *n*_abs(loss)_ + 1. The equations for the off-diagonal terms are decoupled from the populations and we assume that the off-diagonal terms are zero. The heat currents, eqn (2), at steady state are found using eqn (7) and (13),14
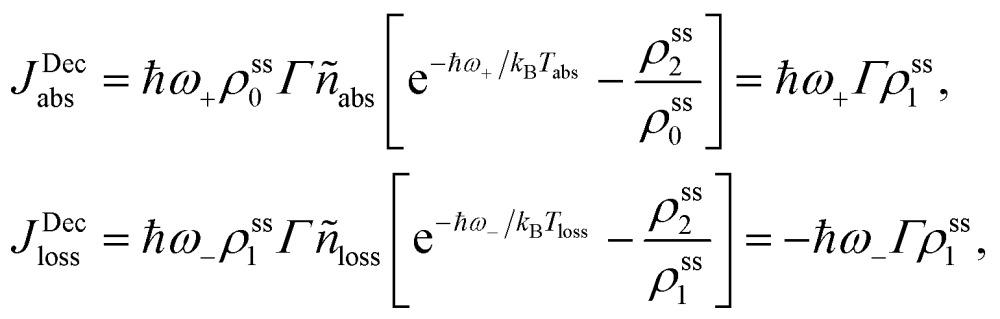
where *ρ*ss*i* are the steady state populations. They are found by setting eqn (13) to zero. The excitation rate to the RC/circuit is *Γρ*ss1, and the power is15
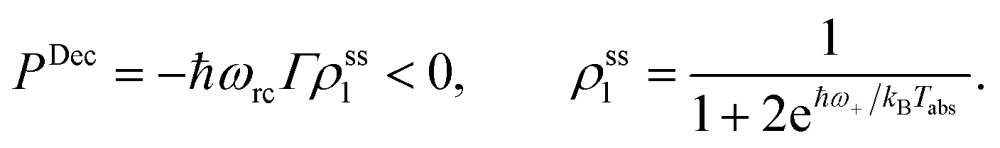



Power is always extracted (*P* ^Dec^ < 0), even if the temperatures are the same, *T*_abs_ = *T*_loss_. This is in contradiction with thermodynamics, which forbids cyclic power extraction in the presence of a single temperature. In combination, the temperature independence of the heat currents ratio and the positivity of *J*Decabs (eqn (3) and (4)), provides further evidence of the violation of thermodynamics,16
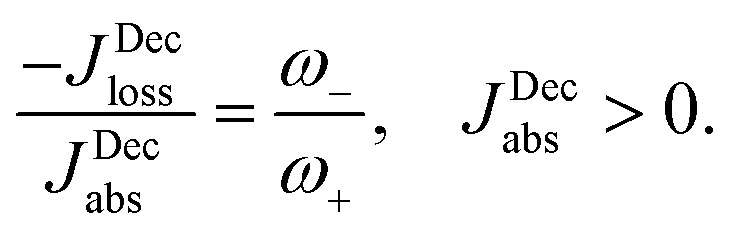



For 
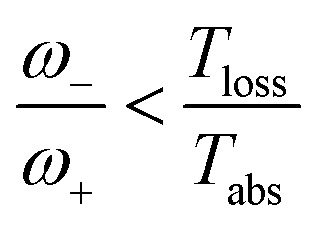
 the model breaks the second law of thermodynamics, eqn (3).

(ii) *Hamiltonian transfer*. For this case the evolution equations in the interaction picture for *ρ*_2,*n*_, *ρ*_+,*n*_ and *ρ*_–,*n*_ are (see ESI-IIB†)17
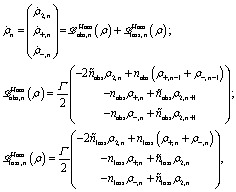
which show a different dynamics compared to the decay transfer scheme (see eqn (13)). From eqn (17) we obtain the power extraction for the Hamiltonian transfer which differs from *P* ^Dec^ (see ESI-IIB†),18
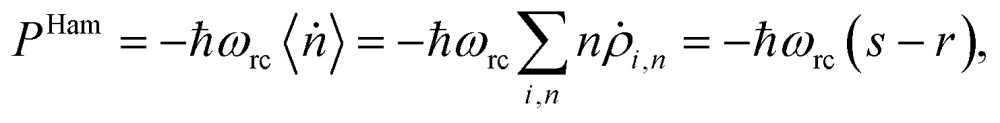

**Fig. 4:**
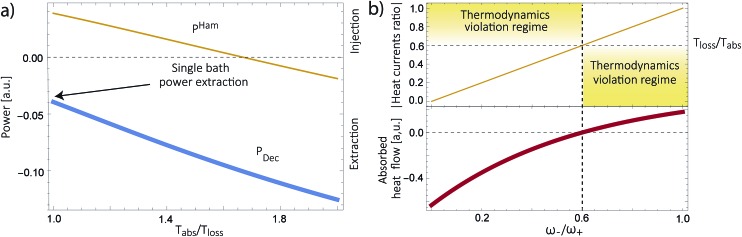
(a) Predicted power extraction for the decay (*P* ^Dec^, thick blue line) and the Hamiltonian (*P* ^Ham^, thin yellow line) transfer schemes. (b) Absolute value of the heat currents ratio (yellow thin line) for the Hamiltonian transfer scheme (top) and *J*Hamabs, (bottom, thick red line).

** is the HO population change. We have assumed an ideal case, where all the energy flow to the HO is considered as power, which just represents a maximum bound.^17,18^ The heat currents, eqn (2), at steady state are found using eqn (11) and (17),

19 *J*Hamabs = *ℏω*_+_(*s* – *r*); *J*Hamloss = –*ℏω*_–_(*s* – *r*),

where *s* – *r* = *K*_1_(e^–*ℏω*_+_/*k*_B_*T*_abs_^ – e^–*ℏω*_–_/*k*_B_*T*_loss_^). *K*_1_ is always positive and depends on the couplings to baths (see ESI-IIB†). In contrast to the decay transfer scheme, in this case power is extracted, *P* ^Ham^ < 0, only for certain combination of parameters,20
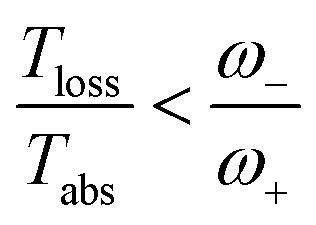
and power can not be extracted if both temperatures are the same. Further divergences between *P* ^Dec^ and *P* ^Ham^ can be seen in Fig. 4a.

Fig. 4b shows the heat currents ratio of the Hamiltonian transfer scheme, which complies with the second law of thermodynamics (see eqn (3)). The thermodynamic violation regime splits due to the *J*Hamabs sign change. Although for positive *J*Hamabs the absolute value of the heat currents ratio should be larger than the temperatures ratio, for negative *J*Hamabs it should be smaller. The lack of sign change for *J*Decabs prevents the splitting of the thermodynamic violation regime, placing the heat currents ratio in a thermodynamically forbidden region (see Fig. 2a).

## Conclusions

We have analyzed several models used for describing energy absorption and transmission both in solar cells and in photosynthetic systems, such as the FMO complex. We have shown that the use of sinks, traps or any artificial relaxation process in order to describe the energy transfer to a further stage (the reaction center in photosynthetic systems or the electric circuit in a solar cell) introduces a contradiction with the second law of thermodynamics. This invalidates several models currently used to study solar energy conversion, casting doubts regarding their conclusions. These include the role of coherences, environment assisted quantum transport, coherent nuclear motion and the presence of quantum effects in photosynthesis, among others. We do not argue against the existence of those effects in the conversion of solar energy. But they should be verified using thermodynamically consistent models.

We have further proposed how to correctly analyze these systems. We show this in a thermodynamically consistent toy model that explicitly describes parts of the RC/circuit and uses a Hamiltonian term to describe the energy transfer instead of a decay rate. The predicted transmitted energy greatly differs between these two alternatives (see Fig. 4a), highlighting the need to review the conclusions derived by thermodynamically inconsistent models.
