# Supplementary material for: On thermodynamic inconsistencies in several photosynthetic and solar cell models and how to fix them
Source: Chem Sci. 2016 Oct 26;8(2):1008–14. doi: 10.1039/c6sc04350j (PMC5354066; doi:10.1039/c6sc04350j)
Supplement: Supplementary file 1 [file SC-008-C6SC04350J-s001.pdf]

Electronic Supplementary Material (ESI) for Chemical Science.
This journal is © The Royal Society of Chemistry 2016

Supplementary Information: On thermodynamic inconsistencies in several photosynthetic and solar cell models and how to fix them

David Gelbwaser-Klimovsky and Alán Aspuru-Guzik
Department of Chemistry and Chemical Biology, Harvard University, Cambridge, MA 02138

I. Energy conversion models

We derive the evolution equations for some examples of two types of energy conversion models. The results of this section are used to generate Figure 2 in the main text, as well as Eq. 3.

IA. Donor-acceptor models

As examples of these models, we analyze below two particular donor-acceptor models that use a decay transfer scheme. This kind of analysis may be expanded to models that include coherent vibronic evolution such as proposed in reference [1].

1) We consider the biological quantum heat engine model proposed in [2] (see in particular Eqs. S34-S37 in reference [2]). It consists of a four level system coupled to a hot bath, a cold bath, and to the reaction center/circuit (also termed "the load"). $T_{h(c)}$ is the hot (cold) bath temperature. The different decay rates are shown in Figure S1a. The equations of motion are

$$\begin{gathered}\dot{\rho}_{aa} = -\gamma_c\left[(1+\bar{n}_c)\,\rho_{aa} - \bar{n}_c\rho_{\alpha\alpha}\right] - \gamma_h\left[(1+\bar{n}_h)\,\rho_{aa} - \bar{n}_h\rho_{bb}\right],\\ \dot{\rho}_{\alpha\alpha} = \gamma_c\left[(1+\bar{n}_c)\,\rho_{aa} - \bar{n}_c\rho_{\alpha\alpha}\right] - \Gamma\rho_{\alpha\alpha},\\ \dot{\rho}_{bb} = \gamma_h\left[(1+\bar{n}_h)\,\rho_{aa} - \bar{n}_h\rho_{bb}\right] + \Gamma_c\left[\left(1+\bar{N}_c\right)\rho_{\beta\beta} - \bar{N}_c\rho_{bb}\right],\\ \rho_{aa} + \rho_{bb} + \rho_{\alpha\alpha} + \rho_{\beta\beta} = 1,\end{gathered} \tag{S1}$$

where we have kept the original paper notation. ρ_{ii} is the level population of state i and $\bar{n}_i$ or $\bar{N}_i$ are the relevant i- bath mode population. For details on the derivation of Eq. S1, we refer the reader to the original paper [2]. The free Hamiltonian of the four level system is

$$H = \sum_{i\in\{a,b,\alpha,\beta\}} \omega_i |i\rangle\langle i|. \tag{S2}$$

The steady state populations are

$$\frac{\rho^{ss}_{aa}}{\rho^{ss}_{\alpha\alpha}} = \frac{\gamma_c\bar{n}_c + \Gamma}{\gamma_c(\bar{n}_c+1)}, \tag{S3}$$

$$\frac{\rho^{ss}_{bb}}{\rho^{ss}_{\alpha\alpha}} = \frac{\Gamma\left[\gamma_c\left(\bar{n}_c+1\right) + \gamma_h\left(\bar{n}_h+1\right)\right] + \gamma_h\gamma_c\bar{n}_c\left(1+\bar{n}_h\right)}{\gamma_h\bar{n}_h\gamma_c\left(\bar{n}_c+1\right)}, \tag{S4}$$

$$\frac{\rho^{ss}_{aa}}{\rho^{ss}_{bb}} = \frac{\left(\gamma_c\bar{n}_c+\Gamma\right)\gamma_h\bar{n}_h}{\Gamma\left\{\gamma_c\left(\bar{n}_c+1\right) + \gamma_h\left(\bar{n}_h+1\right)\right\} + \gamma_h\gamma_c\bar{n}_c\left(1+\bar{n}_h\right)}, \tag{S5}$$

$$\frac{\rho^{ss}_{\beta\beta}}{\rho^{ss}_{bb}} = e^{-\hbar(\omega_\beta-\omega_b)/k_BT_c} + \frac{\gamma_h\bar{n}_h\Gamma\gamma_c\left(\bar{n}_c+1\right)}{\left(1+\bar{N}_c\right)\Gamma_c\left[\Gamma\left\{\gamma_c\left(\bar{n}_c+1\right)+\gamma_h\left(\bar{n}_h+1\right)\right\} + \gamma_h\gamma_c\bar{n}_c\left(1+\bar{n}_h\right)\right]}. \tag{S6}$$

The evolution induced by the hot bath can be obtained from Eq (S1) by setting $\gamma_c = \Gamma = \Gamma_c = 0$. The evolution induced by the cold bath is obtained by setting in the same equations $\gamma_h = \Gamma = 0$. The heat currents at steady state are (see Eq.2 in the main text) obtained using the induced evolution of each bath and the Hamiltonian (S2),

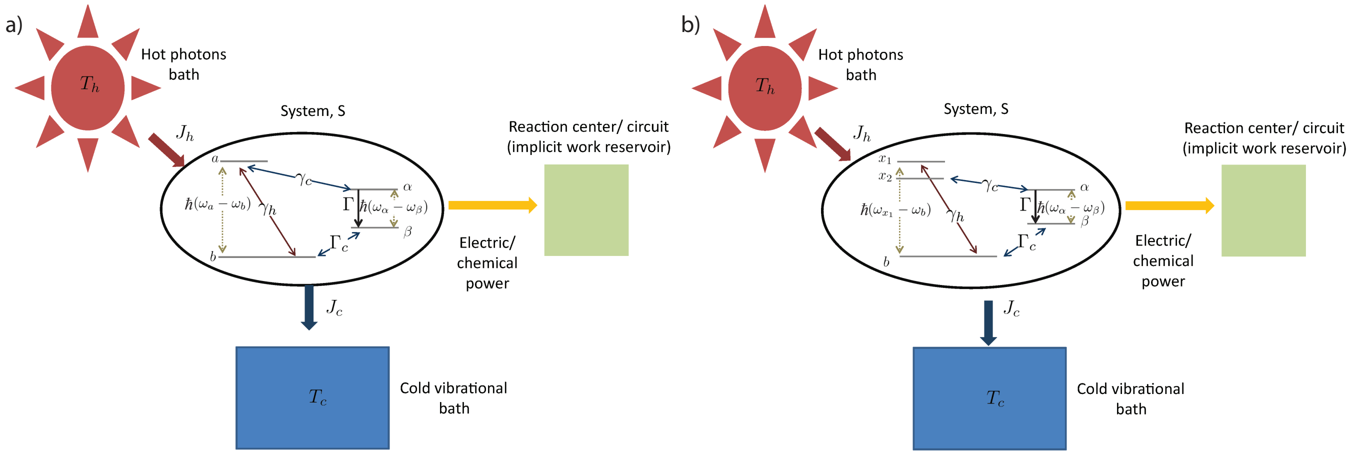

Figure S1: Donor-acceptor models: a) Biological quantum heat engine model from [2]; b) Photocell model proposed in reference [4].

$$J_h = \hbar(\omega_a-\omega_b)\left(1+\bar{n}_h\right)\gamma_h\rho^{ss}_{bb}\left(e^{-\hbar(\omega_a-\omega_b)/k_BT_h}-\frac{\rho^{ss}_{aa}}{\rho^{ss}_{bb}}\right)=$$
$$\frac{\hbar\left(\omega_a-\omega_b\right)\left(1+\bar{n}_h\right)\gamma_h\rho^{ss}_{bb}}{\Gamma\left[\gamma_c\left(\bar{n}_c+1\right)+\gamma_h\left(\bar{n}_h+1\right)\right]+\gamma_h\gamma_c\bar{n}_c\left(1+\bar{n}_h\right)}\left(e^{-\hbar(\omega_a-\omega_b)/k_BT_h}\Gamma\gamma_c(\bar{n}_c+1)\right), \tag{S7}$$

$$J_c=\hbar\left(\omega_a-\omega_\alpha\right)\left(1+\bar{n}_c\right)\gamma_c\rho^{ss}_{\alpha\alpha}\left(e^{-\hbar(\omega_a-\omega_\alpha)/k_BT_c}-\frac{\rho^{ss}_{aa}}{\rho^{ss}_{\alpha\alpha}}\right)+\hbar\left(\omega_\beta-\omega_b\right)\left(1+\bar{N}_c\right)\Gamma_c\rho^{ss}_{bb}\left(e^{-\hbar(\omega_\beta-\omega_b)/k_BT_c}-\frac{\rho^{ss}_{\beta\beta}}{\rho^{ss}_{bb}}\right)=$$
$$-\hbar\left(\omega_a-\omega_\alpha\right)\rho^{ss}_{\alpha\alpha}\Gamma-\hbar\left(\omega_\beta-\omega_b\right)\Gamma_c\left(1+\bar{N}_c\right)\rho^{ss}_{bb}\left(\frac{\gamma_h\bar{n}_h\Gamma\gamma_c\left(\bar{n}_c+1\right)}{\left(1+\bar{N}_c\right)\Gamma_c\left[\Gamma\left\{\gamma_c\left(\bar{n}_c+1\right)+\gamma_h\left(\bar{n}_h+1\right)\right\}+\gamma_h\gamma_c\bar{n}_c\left(1+\bar{n}_h\right)\right]}\right)=$$
$$-\frac{\rho^{ss}_{bb}\gamma_h\bar{n}_h\gamma_c\left(\bar{n}_c+1\right)\Gamma}{\Gamma\left[\gamma_c\left(\bar{n}_c+1\right)+\gamma_h\left(\bar{n}_h+1\right)\right]+\gamma_h\gamma_c\bar{n}_c\left(1+\bar{n}_h\right)}\hbar\left[\omega_a-\omega_\alpha+\omega_\beta-\omega_b\right], \tag{S8}$$

$$-\frac{J_c}{J_h}=\frac{\omega_a-\omega_\alpha+\omega_\beta-\omega_b}{\omega_a-\omega_b}=1+\frac{\omega_\beta-\omega_\alpha}{\omega_a-\omega_b}, \tag{S9}$$

where $\hbar\omega_a-\hbar\omega_b$ $(\hbar\omega_\alpha-\hbar\omega_\beta)$ is the energy of the absorbed (emitted) quanta from the hot bath (to the RC/circuit). Therefore they are equivalent to $\hbar\omega_{abs}(\hbar\omega_{rc})$. Using this paper notation,

$$J_h\rightarrow J_{abs},\quad J_c\rightarrow J_{loss},$$
$$\hbar\omega_\beta-\hbar\omega_\alpha\rightarrow-\hbar\omega_{rc},\quad \hbar\omega_a-\hbar\omega_b\rightarrow\hbar\omega_{abs},$$
$$T_h\rightarrow T_{abs},\quad T_c\rightarrow T_{loss},$$

we obtain Eq. 3 in the main text. A similar analysis can be done for the coherence-assisted biological quantum heat engine model proposed also in the same paper and to the model proposed in reference [3].

2) We consider the photocell model proposed in reference [4]. It consists of a five level system coupled to a hot bath, a cold bath and to the reaction center/circuit (also termed “the load”). $T_{h(c)}$ is the hot (cold) bath temperature. The decay rates are shown in Figure S1b. For the sake of simplicity we assume there is no acceptor-to-donor recombination ($\chi=0$, in the original paper notation). The equations of motion are

$$\begin{aligned}\dot{\rho}_{\alpha\alpha} &= \gamma_c\left[(1+n_{2c})\,\rho_{x2x2} - n_{2c}\rho_{\alpha\alpha}\right] - \Gamma\rho_{\alpha\alpha},\\ \dot{\rho}_{x2x2} &= \gamma_x\left[(1+n_x)\rho_{x1x1} - n_x\rho_{x2x2}\right] - \gamma_c\left[(1+n_{2c})\rho_{x2x2} - n_{2c}\rho_{\alpha\alpha}\right],\\ \dot{\rho}_{bb} &= -\left[\gamma_h n_h + \Gamma_c N_c\right]\rho_{bb} + \gamma_h\,(n_h+1)\,\rho_{x1x1} + \Gamma_c(N_c+1)\rho_{\beta\beta},\\ \dot{\rho}_{x1x1} &= -\gamma_x\left[(1+n_x)\rho_{x1x1} - n_x\rho_{x2x2}\right] - \gamma_h\left[(1+n_h)\rho_{x1x1} - n_h\rho_{bb}\right],\\ \rho_{x1x1} &+ \rho_{x2x2} + \rho_{bb} + \rho_{\alpha\alpha} + \rho_{\beta\beta} = 1.\end{aligned} \tag{S10}$$

where we have kept the original paper notation. ρ_{ii} is the level population of state i and n_i or N_i are the relevant i- bath mode population. For details on the derivation of Eq. S10, we refer the reader to the original paper [4].

The free Hamiltonian of the five level system is

$$H = \sum_{i\in\{x_1,x_2,b,\alpha,\beta\}} \omega_i|i\rangle\langle i|. \tag{S11}$$

The steady state populations are

$$\frac{\rho^{ss}_{x2x2}}{\rho^{ss}_{\alpha\alpha}} = \frac{\Gamma+\gamma_c n_{2c}}{\gamma_c\,(1+n_{2c})}, \tag{S12}$$

$$\frac{\rho^{ss}_{x1x1}}{\rho^{ss}_{x2x2}} = \frac{\gamma_x n_x + \gamma c\,(1+n_{2c}) - \gamma_c n_{2c}\frac{\gamma_c(1+n_{2c})}{\Gamma+\gamma_c n_{2c}}}{\gamma_x\,(1+n_x)} = \frac{\gamma_x n_x\,(\Gamma+\gamma_c n_{2c}) + \gamma c\,(1+n_{2c})\,\Gamma}{\gamma_x\,(1+n_x)\,(\Gamma+\gamma_c n_{2c})}, \tag{S13}$$

$$\frac{\rho^{ss}_{x1x1}}{\rho^{ss}_{bb}} = \frac{\gamma_h n_h\left[\gamma_x n_x\,(\Gamma+\gamma_c n_{2c}) + \gamma_c(1+n_{2c})\Gamma\right]}{\gamma_x\gamma_c\Gamma\,(1+n_x)\,(1+n_{2c}) + \gamma_h\,(1+n_h)\left[\gamma_x n_x\,(\Gamma+\gamma_c n_{2c}) + \gamma_c(1+n_{2c})\Gamma\right]}, \tag{S14}$$

$$\frac{\rho^{ss}_{\beta\beta}}{\rho^{ss}_{bb}} = e^{-\hbar(\omega_\beta-\omega_b)/k_BT_c} + \frac{\gamma_h n_h \gamma_x\gamma_c\Gamma\,(1+n_x)\,(1+n_{2c})}{\Gamma_c\,(1+N_c)\left\{\gamma_x\gamma_c\Gamma\,(1+n_x)\,(1+n_{2c}) + \gamma_h\,(1+n_h)\left[\gamma_x n_x\,(\Gamma+\gamma_c n_{2c}) + \gamma_c\,(1+n_{2c})\,\Gamma\right]\right\}}. \tag{S15}$$

The evolution induced by the hot bath can be obtained from Eq (S10) by setting $\gamma_c = \Gamma = \Gamma_c = \gamma_x = 0$. The evolution induced by the cold bath is obtained by setting in the same equations $\gamma_h = \Gamma = 0$. The heat currents at steady state are (see Eq.2 in the main text) obtained using the induced evolution of each bath and the Hamiltonian (S11),

$$\begin{aligned} J_h &= \hbar\,(\omega_{x1}-\omega_b)\,(1+\bar{n}_h)\,\gamma_h\rho^{ss}_{bb}\left(e^{-\hbar(\omega_{x1}-\omega_b)/k_BT_h} - \frac{\rho^{ss}_{x1x1}}{\rho^{ss}_{bb}}\right) = \\ &\hbar\,(\omega_{x1}-\omega_b)\,\frac{\rho^{ss}_{bb}\gamma_h n_h\gamma_x\gamma_c\Gamma\,(1+n_x)\,(1+n_{2c})}{\gamma_x\gamma_c\Gamma\,(1+n_x)\,(1+n_{2c}) + \gamma_h\,(1+n_h)\left[\gamma_x n_x\,(\Gamma+\gamma_c n_{2c}) + \gamma_c\,(1+n_{2c})\,\Gamma\right]}, \end{aligned} \tag{S16}$$

$$\begin{aligned} J_c &= \hbar\,(\omega_{x1}-\omega_{x2})\,(1+\bar{n}_x)\,\gamma_x\rho^{ss}_{x2x2}\left(e^{-\hbar(\omega_{x1}-\omega_{x2})/k_BT_c} - \frac{\rho^{ss}_{x1x1}}{\rho^{ss}_{x2x2}}\right) + \\ &\hbar\,(\omega_{x2}-\omega_\alpha)\,(1+\bar{n}_{2c})\,\gamma_c\rho^{ss}_{\alpha\alpha}\left(e^{-\hbar(\omega_{x2}-\omega_\alpha)/k_BT_c} - \frac{\rho^{ss}_{x2x2}}{\rho^{ss}_{\alpha\alpha}}\right) + \hbar\,(\omega_\beta-\omega_b)\left(1+\bar{N}_c\right)\Gamma_c\rho^{ss}_{bb}\left(e^{-\hbar(\omega_\beta-\omega_b)/k_BT_c} - \frac{\rho^{ss}_{\beta\beta}}{\rho^{ss}_{bb}}\right) = \\ &-\frac{\rho^{ss}_{bb}\gamma_h n_h\gamma_x\gamma_c\Gamma\,(1+n_x)\,(1+n_{2c})}{\gamma_x\gamma_c\Gamma\,(1+n_x)\,(1+n_{2c}) + \gamma_h\,(1+n_h)\left[\gamma_x n_x\,(\Gamma+\gamma_c n_{2c}) + \gamma_c\,(1+n_{2c})\,\Gamma\right]}\hbar\,(\omega_{x1}+\omega_\beta-\omega_\alpha-\omega_b)\,, \end{aligned} \tag{S17}$$

$$\frac{-J_c}{J_h} = \frac{\omega_{x1}+\omega_\beta-\omega_\alpha-\omega_b}{\omega_{x1}-\omega_b} = 1 + \frac{\omega_\beta-\omega_\alpha}{\omega_{x1}-\omega_b}, \tag{S18}$$

where $\hbar\omega_{x1} - \hbar\omega_b$ ($\hbar\omega_\alpha - \hbar\omega_\beta$) is the energy of the absorbed (emitted) quanta from the hot bath (to the RC/circuit), therefore equivalent to $\hbar\omega_{abs}(\hbar\omega_{rc})$. Using this paper notation,

$$J_h \to J_{abs}, \quad J_c \to J_{loss},$$
$$\hbar\omega_\beta - \hbar\omega_\alpha \to -\hbar\omega_{rc}, \quad \hbar\omega_{x1} - \hbar\omega_b \to \hbar\omega_{abs},$$
$$T_h \to T_{abs}, \quad T_c \to T_{loss},$$

we obtain Eq. 3 in the main text.

IB. Standard FMO models

We use the standard model proposed in reference [5] for the Fenna-Mathews-Olson (FMO) complex of the green sulfur bacterium *Prosthecochloris aestuarii*. Its dynamics is governed by the following Hamiltonian,

$$H_{FMO} = H_{sites} + H_{FMO-vib} + H_{vib} + H_{Dec}, \tag{S19}$$

where H_{vib} is the free Hamiltonian for the vibrational degrees of freedom of the pigments and proteins, which we assume to be at equilibrium at a temperature $T_{loss} = 300K$. H_{sites} is the exciton Hamiltonian,

$$H_{sites} = \sum_{m\in FMO} E_m|m\rangle\langle m| + \sum_{m\neq n\in FMO} V_{mn}|m\rangle\langle n|, \tag{S20}$$

where $|m\rangle$ is the excited state of the m site, and the sum is over all the FMO sites. $H_{FMO-vib}$ represents the interaction between the excitons and the vibrational bath,

$$H_{FMO-vib} = \sum_{m\in FMO,\xi} k_\xi^m|m\rangle\langle m| \otimes Q_\xi, \tag{S21}$$

where Q_ξ operates on the vibrational degrees of freedom. All the parameters for this Hamiltonian can be found in reference [5].

Transmission of energy to the reaction center

The Hamiltonian H_{Dec} governs the transmission of energy to the reaction center and is typically modeled [1, 6–12] as an irreversible decay term from the FMO site 3 to 8,

$$H_{Dec} = \sqrt{\Gamma_{3,8}}|8\rangle\langle 3|. \tag{S22}$$

We use a typical value for this rate, $\Gamma_{3,8} = 62.8/1.88\,cm^{-1}$[6–8, 12].

Thermal radiation

Even though at the surface of the Sun the emitted thermal radiation is at thermal equilibrium with the same temperature as the Sun, T_S, due to geometric considerations, only a small fraction of those photons reaches the Earth. This is quantified by a geometric factor $\lambda = 2 * 10^{-5}$ equal to the angle subtended by the Sun seen from the Earth. If $n_{T_S}[\omega]$ photons of frequency ω are emitted from the Sun, only λn_{T_s} reach the Earth. This radiation is no longer at thermal equilibrium, but rather is a non-equilibrium bath at an effective temperature [13–15],

$$e^{-\hbar\omega_{ant}/k_B T_{abs}} = \frac{\lambda n_{T_S}[\omega_{ant}]}{\lambda n_{T_S}[\omega_{ant}] + 1} \to T_{abs} \sim 1356K. \tag{S23}$$

where $n_{T_S}[\omega] = (e^{\hbar\omega/k_B T_S} - 1)^{-1}$. The dilution of the photon numbers turns the effective temperature, T_{abs}, frequency dependent. Nevertheless, the frequency variation between the antenna and the FMO site is small, therefore we assume the same T_{abs} for the antenna and the FMO sites.

II. Dynamic equations for simple models for the RC/circuit

In this section, we explain the preliminary steps taken for deriving the dynamics of the simple models for the RC/circuit (Eqs. 8 and 12 in the main text).

We consider a three level system (3LS), S, coupled to the reaction center (RC) or electric circuit. The later is a reservoir of independent quinones/sites, each of them represented by a single two level system (TLS). Its ground state represents an empty quinone/site and the excited state corresponds to a full quinone/site. Besides, the 3LS is coupled to a photon (hot) bath and a vibrational (cold) bath (see Figure 3 in the main text). The total Hamiltonian is

$$H_S + H_B + H_{SB}, \tag{S24}$$

where $H_B = H_{photons} + H_{phonons}$ is the baths free Hamiltonian. The S-baths coupling Hamiltonian is given by

$$H_{SB} = S \otimes (B_h + B_c) = \sum_\lambda g_{h,\lambda} \left(|2\rangle\langle 0| a_\lambda + |0\rangle\langle 2| a_\lambda^\dagger \right) + \sum_\lambda g_{c,\lambda} \left(|2\rangle\langle 1| b_\lambda + |1\rangle\langle 2| b_\lambda^\dagger \right), \tag{S25}$$

where a_λ,$a_\lambda^\dagger$ (b_λ, $b_\lambda^\dagger$) are the annihilation and creation operators of photons (phonons) modes. The S + RC/circuit Hamiltonian is

$$H_S = H_0 + H_{trans}, \qquad H_0 = \hbar\omega_{abs}|2\rangle\langle 2| + \frac{\hbar\omega_{rc}}{2} \left(|1\rangle\langle 1| - |0\rangle\langle 0| \right), \tag{S26}$$

where H_0 is the 3LS free Hamiltonian and H_{trans} describes the energy transfer to the RC/circuit. We compare between two possible schemes: i) a decay transfer described by a non-Hermitian H_{trans}; ii) a Hamiltonian transfer, represented by a Hermitian H_{trans}.

IIA. Decay transfer

The decay transfer is described by the following non-Hermitian term,

$$H^{Dec}_{tranf} = \sqrt{\Gamma}|0\rangle\langle 1|. \tag{S27}$$

As a first step we transform the S-bath interaction and the transfer Hamiltonian to the interaction picture

$$H_{SB} \to e^{iH_0 t} H_{SB} e^{-iH_0 t}, \qquad H^{Dec}_{tranf} \to e^{iH_0 t} H^{Dec}_{tranf} e^{-iH_0 t}. \tag{S28}$$

H^{Dec}_{tranf} is a fictitious Hamiltonian due to its lack of Hermiticity, therefore cannot form part of the rotation, $e^{iH_0 t}$, which has to be unitary. Besides, we derive the reduced dynamics only for S. The operators in the interaction picture are:

$$|2\rangle\langle 0|\,[t] = e^{it\hbar\left(\omega_{abs} + \frac{\omega_{rc}}{2}\right)}|2\rangle\langle 0|, \qquad |2\rangle\langle 1|\,[t] = e^{it\hbar\left(\omega_{abs} - \frac{\omega_{rc}}{2}\right)}|2\rangle\langle 1|, \qquad |0\rangle\langle 1|\,[t] = e^{-it\hbar\omega_{rec}}|0\rangle\langle 1|. \tag{S29}$$

Using the standard Born-Markov approximation, the Lindblad equation [16] for S is obtained (Eq.8 in the main text). The steady state populations are

$$\frac{\rho^{ss}_{22}}{\rho^{ss}_{11}} = 1, \qquad \frac{\rho^{ss}_{22}}{\rho^{ss}_{00}} = e^{-\hbar\omega_+/k_B T_{abs}} - \frac{\rho^{ss}_{11}}{\widetilde{n}_{abs}\rho^{ss}_{00}}, \qquad \frac{\rho^{ss}_{11}}{\rho^{ss}_{00}} = \frac{n_{abs}\widetilde{n}_{loss}}{n_{loss}\widetilde{n}_{abs} + \widetilde{n}_{abs} + \widetilde{n}_{loss}},$$
$$\rho^{ss}_{11} = \frac{1}{1 + \frac{\rho^{ss}_{00}}{\rho^{ss}_{11}} + \frac{\rho^{ss}_{22}}{\rho^{ss}_{11}}} = \frac{1}{1 + 2e^{\hbar\omega_+/k_B T_{abs}}}. \tag{S30}$$

where $n_{abs(loss)}$ is the photon (vibrational) bath population of mode $\omega_\pm = \omega_{abs} \pm \frac{\omega_{rc}}{2}$ and $\widetilde{n}_{abs(loss)} = n_{abs(loss)} + 1$.

IIB. Hamiltonian transfer

Here we explicitly consider the RC/circuit and its coupling to S, by considering H_{trans} as an Hermitian Hamiltonian. The RC/circuit is composed of identical and independent TLSs. The S + RC/circuit Hamiltonian is:

$$H_S = \hbar\omega_{abs}|2\rangle\langle 2| + \frac{\hbar\omega_{rc}}{2}\left(|1\rangle\langle 1| - |0\rangle\langle 0|\right) + \sum_k^j \sqrt{\frac{\Gamma}{2j}}\left(\sigma_-^k|1\rangle\langle 0| + \sigma_+^k|0\rangle\langle 1|\right) + \hbar\omega_{rc}\sum_k \sigma_z^k, \tag{S31}$$

where j is the number of TLSs.

In order to find the energy that is being transferred to the RC/circuit, we start by diagonalizing the S + RC circuit. This is achieved by first applying the Holstein-Primakoff transformation [17], that consists on the introduction of the following collective operators:

$$\sum_k \sigma_-^k = \left(\sqrt{2j - c^\dagger c}\right)c, \qquad \sum_k \sigma_+^k = c^\dagger\left(\sqrt{2j - c^\dagger c}\right), \qquad \sum_k \sigma_z^k = c^\dagger c - j. \tag{S32}$$

The new Hamiltonian is

$$H_S = \hbar\omega_{abs}|2\rangle\langle 2| + \frac{\hbar\omega_{rc}}{2}\left(|1\rangle\langle 1| - |0\rangle\langle 0|\right) + \sqrt{\frac{\Gamma}{2j}}\left[\left(\sqrt{2j - c^\dagger c}\right)c|1\rangle\langle 0| + c^\dagger\left(\sqrt{2j - c^\dagger c}\right)|0\rangle\langle 1|\right] + \hbar\omega_{rc}\left(c^\dagger c - j\right). \tag{S33}$$

At this point, the modes are displaced, $c \to c - \sqrt{\epsilon}$,

$$H_S = \hbar\omega_{abs}|2\rangle\langle 2| + \frac{\hbar\omega_{rc}}{2}\left(|1\rangle\langle 1| - |0\rangle\langle 0|\right) + \sqrt{\frac{\Gamma k\eta}{2j}}\left(c|1\rangle\langle 0| + c^\dagger|0\rangle\langle 1|\right) - \sqrt{\frac{\Gamma k\eta\epsilon}{2j}}\left(|1\rangle\langle 0| + |0\rangle\langle 1|\right) + \hbar\omega_{rc}\left(c^\dagger c - \sqrt{\epsilon}(c + c^\dagger) + \epsilon - j\right), \tag{S34}$$

where $k = 2j - \epsilon$ and $\eta = 1 - \frac{c^\dagger c - \sqrt{\epsilon}(c^\dagger + c)}{k}$. We assume that the number of TLSs is large, $\frac{c^\dagger c - \sqrt{\epsilon}(c^\dagger + c)}{k} \ll 1$. The physical interpretation of this approximation is clarified below. Under this assumptions, we expand $\sqrt{\eta} \approx 1 - \frac{c^\dagger c - \sqrt{\epsilon}(c^\dagger + c)}{2k} - \frac{\epsilon(c^\dagger + c)^2}{8k^2}$ and keep terms up to order $\frac{1}{\sqrt{j}}$,

$$\begin{aligned} H_S = \hbar\omega_{abs}|2\rangle\langle 2| + \frac{\omega_{rc}}{2}\left(|1\rangle\langle 1| - |0\rangle\langle 0|\right) - \sqrt{\frac{\Gamma k\epsilon}{2j}}\left(|1\rangle\langle 0| + |0\rangle\langle 1|\right) + \sqrt{\frac{\Gamma k}{2j}}\left(c|1\rangle\langle 0| + c^\dagger|0\rangle\langle 1|\right) - \\ \frac{\epsilon}{2}\sqrt{\frac{\Gamma}{2jk}}\left(c^\dagger + c\right)\left(|1\rangle\langle 0| + |0\rangle\langle 1|\right) + \hbar\omega_{rc}\left(c^\dagger c - \sqrt{\epsilon}\left(c + c^\dagger\right) + \epsilon - j\right). \end{aligned} \tag{S35}$$

Setting $\epsilon = 0$, the Hamiltonian is simplified to

$$H_S = \hbar\omega_{abs}|2\rangle\langle 2| + \frac{\hbar\omega_{rc}}{2}\left(|1\rangle\langle 1| - |0\rangle\langle 0|\right) + \sqrt{\Gamma}\left(c|1\rangle\langle 0| + c^\dagger|0\rangle\langle 1|\right) + \hbar\omega_{rc}\left(c^\dagger c - j\right) \tag{S36}$$

and the approximation to $\frac{c^\dagger c}{2j} \ll 1$. Therefore, we are just assuming that the total number of excitations in the RC/circuit is very small compared to the number of quinones/sites, so energy may always be transferred to the RC/circuit. From Eq. S36, we derive Eq. 8 in the main text,

$$H_{trasns}^{Ham} = \sqrt{\Gamma}\left(c|1\rangle\langle 0| + c^\dagger|0\rangle\langle 1|\right) + \hbar\omega_{rc}\left(c^\dagger c - j\right). \tag{S37}$$

which is an effective equation for a harmonic oscillator (HO) coupled to S. Therefore, from now on we model the RC/circuit as a HO. Next we diagonalize Eq. S36. The Hamiltonian eigenvectors are

$$|+,n\rangle = \frac{1}{\sqrt{2}}\left(|1,n\rangle + |0,n+1\rangle\right), \qquad |-,n\rangle = \frac{1}{\sqrt{2}}\left(|0,n+1\rangle - |1,n\rangle\right), \tag{S38}$$

$$E_{\pm} = \hbar\omega_{rc}\left(n+\frac{1}{2}\right) \pm \frac{\hbar\Omega_n}{2} - j\hbar\omega_{rc}, \tag{S39}$$

$$H_S + H_{trans}^{Ham} = \hbar\omega_{abs}|2,n\rangle\langle 2,n| + \hbar\omega_{rc}\left(\tilde{c}^{\dagger}\tilde{c} - j\right) + \sum_n \frac{\hbar\omega_{rc}}{2}\left\{\left(1+\frac{\Omega_n}{\omega_{rc}}\right)|+,n\rangle\langle +,n| + \left(1-\frac{\Omega_n}{\omega_{rc}}\right)|-,n\rangle\langle -,n|\right\}, \tag{S40}$$

where $\tilde{c}^{\dagger}(\tilde{c})$ is the creation (annihilation) operator in the new basis and $\Omega_n = 2\sqrt{\Gamma(n+1)}$. The inverse transformations are

$$|1,n\rangle = \frac{1}{\sqrt{2}}\left(|+,n\rangle - |-,n\rangle\right), \qquad |0,n+1\rangle = \frac{1}{\sqrt{2}}\left(|+,n\rangle + |-,n\rangle\right). \tag{S41}$$

Rewriting the S-bath Hamiltonian, Eq. S25 , in the new basis,

$$|2\rangle\langle 0| = \sum_n \frac{1}{\sqrt{2}}\left(|2,n+1\rangle\langle +,n| + |2,n+1\rangle\langle -,n|\right), \qquad |2\rangle\langle 1| = \sum_n \frac{1}{\sqrt{2}}\left(|2,n\rangle\langle +,n| - |2,n\rangle\langle -,n|\right), \tag{S42}$$

and transforming to the interaction picture,

$$H_{SB} \to e^{iH_S t} H_{SB} e^{-iH_S t},$$
$$|2\rangle\langle 0|\,[t] = \sum_n \frac{1}{\sqrt{2}}\left(e^{it\hbar\left(\omega_+ - \frac{\Omega_n}{2}\right)}|2,n+1\rangle\langle +,n| + e^{it\hbar\left(\omega_+ + \frac{\Omega_n}{2}\right)}|2,n+1\rangle\langle -,n|\right),$$
$$|2\rangle\langle 1|\,[t] = \sum_n \frac{1}{\sqrt{2}}\left(e^{it\hbar\left(\omega_- - \frac{\Omega_n}{2}\right)}|2,n\rangle\langle +,n| - e^{it\hbar\left(\omega_- + \frac{\Omega_n}{2}\right)}|2,n\rangle\langle -,n|\right). \tag{S43}$$

In contrast to the decay transfer scheme (Eq. S28), here H_{trans}^{Ham} is Hermitian and we derive the reduced dynamics for the S + RC/circuit. Therefore H_{trans}^{Ham} is included in the rotation, $e^{iH_S t}$. Using the standard Born-Markov approximation, the Lindblad equation [16] for S + RC/circuit is obtained, and from it the evolution equations are derived,

$$\dot{\rho}_{+,n} = \frac{\Gamma}{2}\left\{-\left(n_{abs}\left[\omega_+ - \frac{\Omega_n}{2}\right] + n_{loss}\left[\omega_- - \frac{\Omega_n}{2}\right]\right)\rho_{+,n} + \widetilde{n}_{loss}\left[\omega_- - \frac{\Omega_n}{2}\right]\rho_{2,n} + \widetilde{n}_{abs}\left[\omega_+ - \frac{\Omega_n}{2}\right]\rho_{2,n+1}\right\},$$
$$\dot{\rho}_{-,n} = \frac{\Gamma}{2}\left\{-\left(n_{abs}\left[\omega_+ + \frac{\Omega_n}{2}\right] + n_{loss}\left[\omega_- + \frac{\Omega_n}{2}\right]\right)\rho_{-,n} + \widetilde{n}_{loss}\left[\omega_- + \frac{\Omega_n}{2}\right]\rho_{2,n} + \widetilde{n}_{abs}\left[\omega_+ + \frac{\Omega_n}{2}\right]\rho_{2,n+1}\right\},$$
$$\dot{\rho}_{2,n} = \frac{\Gamma}{2}\left\{-\left(\widetilde{n}_{abs}\left[\omega_+ - \frac{\Omega_{n-1}}{2}\right] + \widetilde{n}_{abs}\left[\omega_+ + \frac{\Omega_{n-1}}{2}\right] + \widetilde{n}_{loss}\left[\omega_- + \frac{\Omega_n}{2}\right] + \widetilde{n}_{loss}\left[\omega_- - \frac{\Omega_n}{2}\right]\right)\rho_{2,n}\right.$$
$$\left. n_{loss}\left[\omega_- - \frac{\Omega_n}{2}\right]\rho_{+,n} + n_{loss}\left[\omega_- + \frac{\Omega_n}{2}\right]\rho_{-,n} + n_{abs}\left[\omega_+ - \frac{\Omega_{n-1}}{2}\right]\rho_{+,n-1} + n_{abs}\left[\omega_+ + \frac{\Omega_{n-1}}{2}\right]\rho_{-,n-1}\right\}, \tag{S44}$$

where ρ_i is the population of the combined state i (S + RC/circuit), Γ_i and $n_i\,[\omega]$ are the decay rate and the ω-mode population of the i-bath, respectively, and $\widetilde{n}_i\,[\omega] = n_i\,[\omega] + 1$. The equations for the off-diagonal terms are decoupled from the populations and for simplicity we assume that the off-diagonal terms are zero. In order to simplify the evolution equations we assume that the mode population does not change on shift of the order of Ω_n, therefore $n_i\left[\omega_\pm \pm \frac{\Omega_n}{2}\right] \approx n_i\,[\omega_\pm]$. The resulting equations are shown in the main text, Eqs. 12.

The 3LS steady state is obtained by summing over n Eqs. 12 in the main text. It is

$$\frac{\rho_2^{ss}}{\rho_+^{ss}} = \frac{n_{abs} + n_{loss}}{\widetilde{n}_{abs} + \widetilde{n}_{loss}}, \quad \rho_+^{ss} = \frac{\widetilde{n}_{abs} + \widetilde{n}_{loss}}{3\widetilde{n}_{abs} + 3\widetilde{n}_{loss} - 2}, \tag{S45}$$

where $n_{abs(loss)}$ is the photon (vibrational) bath population of mode $\omega_\pm = \omega_{abs} \pm \frac{\omega_{rc}}{2}$. Using these expressions, we can find the evolution for the HO excitation energy,

$$\hbar\omega_{rc}\langle\dot{n}\rangle = \hbar\omega_{rc}\,(s-r)\,, \tag{S46}$$

which is equal to $-P^{Ham}$ (the used sign convention can be found below Eq. 1 in the main text). Thus, $s > r$ is required in order to increase the RC/circuit energy. At the 3LS steady state, this implies,

$$s - r = \frac{\Gamma\widetilde{n}_{loss}\widetilde{n}_{abs}}{3\widetilde{n}_{abs} + 3\widetilde{n}_{loss} - 2}\left(e^{-\hbar\omega_{+}/k_B T_{abs}} - e^{-\hbar\omega_{-}/k_B T_{loss}}\right) = K_1\left(e^{-\hbar\omega_{+}/k_B T_{abs}} - e^{-\hbar\omega_{-}/k_B T_{loss}}\right) > 0, \tag{S47}$$

where $K_1 = \frac{\Gamma\widetilde{n}_{loss}\widetilde{n}}{3\widetilde{n}_h + 3\widetilde{n}_c - 2} > 0$ and the energy gain condition is

$$\frac{T_{loss}}{T_{abs}} < \frac{\omega_{-}}{\omega_{+}}. \tag{S48}$$

[1] N. Killoran, S. F. Huelga and M. B. Plenio, *arXiv preprint arXiv:1412.4136*, 2014.
[2] K. E. Dorfman, D. V. Voronine, S. Mukamel and M. O. Scully, *Proceedings of the National Academy of Sciences*, 2013, **110**, 2746–2751.
[3] M. O. Scully, K. R. Chapin, K. E. Dorfman, M. B. Kim and A. Svidzinsky, *Proceedings of the National Academy of Sciences*, 2011, **108**, 15097–15100.
[4] C. Creatore, M. Parker, S. Emmott and A. Chin, *Phys. Rev. Lett.*, 2013, **111**, 253601.
[5] J. Adolphs and T. Renger, *Biophys. J.*, 2006, **91**, 2778–2797.
[6] M. Mohseni, P. Rebentrost, S. Lloyd and A. Aspuru-Guzik, *The Journal of chemical physics*, 2008, **129**, 174106.
[7] P. Rebentrost, M. Mohseni, I. Kassal, S. Lloyd and A. Aspuru-Guzik, *New Journal of Physics*, 2009, **11**, 033003.
[8] M. B. Plenio and S. F. Huelga, *New Journal of Physics*, 2008, **10**, 113019.
[9] Y. Dubi, *The Journal of Physical Chemistry C*, 2015, **119**, 25252–25259.
[10] F. H. Alharbi and S. Kais, *Renewable and Sustainable Energy Reviews*, 2015, **43**, 1073–1089.
[11] J. Cao and R. J. Silbey, *J. Phys. Chem. A*, 2009, **113**, 13825–13838.
[12] F. Caruso, A. W. Chin, A. Datta, S. F. Huelga and M. B. Plenio, *The Journal of Chemical Physics*, 2009, **131**, 105106.
[13] P. Würfel and U. Würfel, *Physics of solar cells: from basic principles to advanced concepts*, John Wiley & Sons, 2009.
[14] P. Landsberg and G. Tonge, *J. Appl. Phys.*, 1980, **51**, R1–R20.
[15] R. Alicki and D. Gelbwaser-Klimovsky, *New Journal of Physics*, 2015, **17**, 115012.
[16] G. Lindblad, *Communications in Mathematical Physics*, 1976, **48**, 119–130.
[17] T. Holstein and H. Primakoff, *Phys. Rev.*, 1940, **58**, 1098.
